# Recent trends in prenatal genetic screening and testing

**DOI:** 10.12688/f1000research.16837.1

**Published:** 2019-05-31

**Authors:** Ondrej Pös, Jaroslav Budiš, Tomáš Szemes

**Affiliations:** 1 Faculty of Natural Sciences, Comenius University, Bratislava, 84215, Slovakia; 2 University Science Park, Comenius University, Bratislava, 84104, Slovakia

**Keywords:** NIPT, fetal aneuploidies, amniocentesis, screening, cffDNA, non-invasive

## Abstract

Prenatal testing in recent years has been moving toward non-invasive methods to determine the fetal risk for genetic disorders without incurring the risk of miscarriage. Rapid progress of modern high-throughput molecular technologies along with the discovery of cell-free fetal DNA in maternal plasma led to novel screening methods for fetal chromosomal aneuploidies. Such tests are referred to as non-invasive prenatal tests (NIPTs), non-invasive prenatal screening, or prenatal cell-free DNA screening. Owing to many advantages, the adoption of NIPT in routine clinical practice was very rapid and global. As an example, NIPT has recently become a standard screening procedure for all pregnant women in the Netherlands. On the other hand, invasive sampling procedures remain important, especially for their diagnostic value in the confirmation of NIPT-positive findings and the detection of Mendelian disorders. In this review, we focus on current trends in the field of NIPT and discuss their benefits, drawbacks, and consequences in regard to routine diagnostics.

## Evolution of prenatal testing and diagnosis

**Figure 1.**
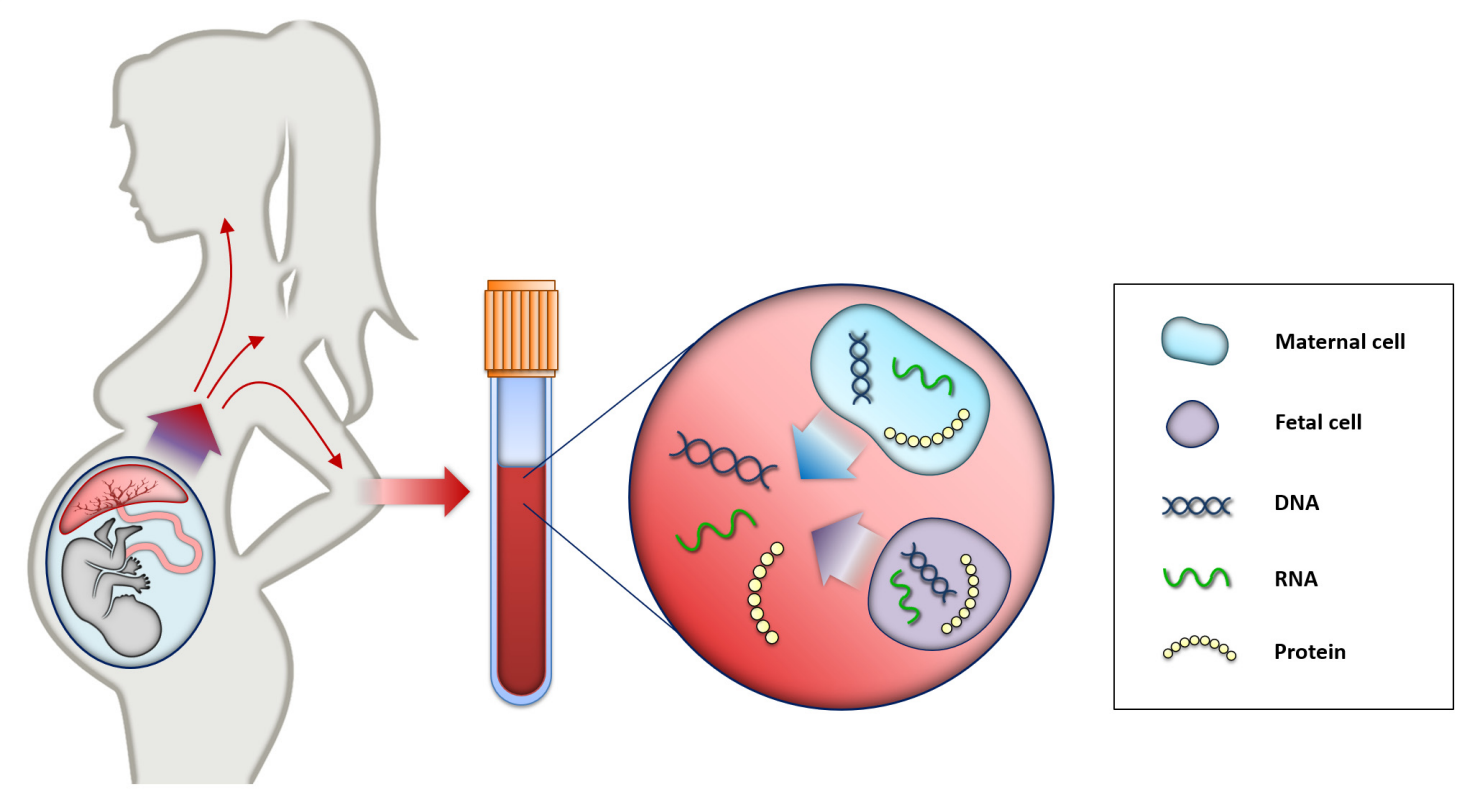
Principle of non-invasive prenatal testing. Maternal blood consists of maternal and placental cells, which release their DNA content directly into maternal circulation. Therefore, cell-free fetal elements (for example, DNA, RNA, and proteins) are present in the blood of pregnant woman and can be used as biomarkers for prenatal testing and diagnosis ^21, 22^.

In current clinical practice, various options of prenatal testing are available for pregnant women in developed countries. However, this convenience has been available only in the last few years. Prenatal testing has passed a long evolution from traditional invasive methods (for example, amniocentesis or chorionic villus sampling [CVS]) ^1^. Early reports of transabdominal amniocentesis came from 1877 but this procedure became more prevalent in the 1970s for genetic diagnosis in high-risk pregnancies ^2, 3^. CVS was first described by Mohr in 1968 ^4^. Since the introduction of ultrasound guidance in 1980, the safety of CVS has increased ^5^, and this method has become widely accepted in routine prenatal diagnosis. A tremendous contribution to prenatal testing was the implementation of non-invasive procedures based on blood sampling ( Figure 1). In 1959, Zipursky *et al*. found that intact fetal cells are present in maternal plasma ^6^; in 1969, Walknowska *et al*. showed that this approach may have implications for prenatal diagnosis ^7^. However, the main limitation of the method is a low concentration of intact fetal cells in maternal circulation. The detection of cell-free fetal DNA (cffDNA) in maternal plasma, by Lo *et al*. in 1997, launched a new era of non-invasive prenatal testing (NIPT) which has been integrated in clinical practice and represents the standard in developed countries today ^8^. It was shown that a major fraction of cffDNA is released into the maternal circulation during apoptosis of trophoblasts in placenta, which means that, unlike DNA isolated from circulating fetal cells, cffDNA is actually of placental origin ^9^. According to previous calculations, the cffDNA concentration is almost 25 times higher than the concentration of fetal DNA extracted from intact nucleated blood cells in a similar volume of whole maternal blood. Moreover, the approach using cffDNA provides easier, less labor-intensive, and less time-consuming ways to work with fetal DNA ^10^. Current NIPT procedures cannot be performed without modern molecular technologies (for example, next-generation sequencing (NGS)). This is why the cffDNA-based NIPT became commercially available and widespread since 2011 ^11, 12^. Nowadays, NIPT is being implemented in public prenatal care in several countries (for example, the Netherlands) ^13^.

## Cell-free fetal DNA–based approach

Cell-free fragments derived from fetal DNA are shorter than those of maternal cell-free DNA (cfDNA), and the size distribution is typically lower than 150 base pairs ^14, 15^. According to Ashoor *et al*., at 11 to 13 weeks of gestation, the concentration of the fetal DNA fraction ranges from 7.8 to 13.0% depending on gestational age; thus, for analysis of aneuploidy, it is possible to obtain a useful result after 10 weeks of gestation ^16^. In the vast majority of cases, cffDNA is no longer detectable 24 hours after birth and this is due to rapid clearance ^17, 18^. Another factor affecting cffDNA fraction is maternal body mass index. It was shown that the median fetal fraction decreased with maternal weight, from 11.7% at 60 kg to 3.9% at 160 kg ^16^. The relative decrease in fetal fraction could be caused by an increased level of maternal cfDNA originating from active necrosis and apoptosis of adipose tissue in obese pregnant women. The study by Haghiac *et al*. showed that maternal cfDNA levels are elevated in obese compared with lean pregnant women but that cffDNA concentrations remain unchanged ^19^. Because the ratio of fetal fraction decreases with increasing maternal weight, maternal obesity has a negative impact on the diagnostic capability of genetic screening; thus, NIPT is less likely to provide an informative result in obese patients ^20^. Moreover, it was shown that levels of placental proteins—such as free beta human chorionic gonadotropin, pregnancy-associated plasma protein A (PAPP-A), and placental growth factor (PlGF)—are positively correlated with fetal fraction and placental mass. Furthermore, low PAPP-A and PlGF levels correlate with a higher risk of adverse pregnancy outcome, so low fetal fraction may be a useful additional parameter in detecting a high-risk group of pregnancies ^23^.

With increasing throughput and lowering cost, NGS technology became available and, together with cffDNA analysis, opened a new horizon for detection of trisomies and sub-chromosomal aberrations in a non-invasive manner. Current approaches are based on low-coverage massively parallel whole-genome sequencing analysis of plasma DNA from pregnant women. Total cfDNA is sequenced, sequence reads are aligned to reference human genome, and aligned reads are counted ^24^. Thus, a proportional representation of each chromosome can be calculated, and chromosome ploidy status can be determined ^11^.

Studies also reported the use of whole-genome sequencing of plasma DNA for the detection of sub-chromosomal copy number variants (for example, microdeletions and microduplications). However, these approaches are limited by the requirement for an exceptionally high number of sequenced reads and complicated interpretation because of the identification of variants of unknown clinical significance ^25^. Petersen *et al*. estimated lower positive predictive values (0–21%) and higher false-positive rates (79–100%) for the selected microdeletion syndromes (cri du chat/5p- syndrome, Prader–Willi/Angelman syndromes, 22q11del/DiGeorge syndrome, and 1p36 deletion syndrome) compared with common aneuploidies ^26^. Given the low prevalence leading to low positive predictive values, screening for microdeletions should not be used in the general population until clinical validation studies indicate value for the low-risk patients ^27^. Although expanded NIPT screening is already integrated into clinical practice, the American College of Obstetricians and Gynecologists does not recommend routine cffDNA screening for microdeletions at this time ^28^.

While this technology has been widely applied for aneuploidy, there has been relatively little clinical application for the diagnosis of monogenic disorders ^29^. Early diagnosis of monogenic disorders has been challenging because of background maternal cfDNA which prevents direct observation of maternally inherited alleles ^30^. Since these tests are provided on a custom-made basis confined to families at known high risk, there is practically no commercial interest, thus less attention has been given to testing for monogenic disorders ^31^. However, recently, it was shown that it is possible to non-invasively diagnose prenatal monogenic diseases by combining targeted haplotyping of two parents with targeted sequencing of cfDNA extracted during pregnancy ^32^.

The fast adoption of cffDNA-based NIPT to clinical practice reflects its many benefits. It is a non-invasive, relatively painless, and safe procedure without the related risk of miscarriage which is associated with amniocentesis and CVS. On the other hand, there are still a number of samples that cannot be interpreted with certainty. A source of such uninformative results is the nature of the statistical testing. If the standard cutoff threshold for the reliable conclusion of healthy samples is a z-score of 2.5, the chance that a healthy sample would achieve a greater z-score is estimated to be around 1.86% ^33^. Also, biological reasons such as maternal malignancy, fetoplacental mosaicism, or non-identical vanishing twins may contribute to incorrect predictions of the fetal condition ^34– 36^. However, in spite of these limitations, NIPT has been shown to be a highly accurate method for detection of common fetal chromosomal aneuploidies ( Table 1) ^37^. According to clinical validation, the American College of Medical Genetics and Genomics (ACMG) suggests that NIPT can replace conventional screening for Patau, Edwards, and Down syndromes; however, use of the updated ACMG guidelines and statements is recommended to provide quality prenatal care ^38^. However, it should be noted that NIPT is not a diagnostic test and should be confirmed by invasive testing for the presence of any abnormal results ^39^. Even the ultrasound scan is still an important component of first-trimester screening, and the International Society of Ultrasound in Obstetrics and Gynecology recommends ultrasound because it allows clinicians to detect additional structural or chromosomal abnormalities (or both) that may not show up in the blood test ^40^.

**Table 1.** Meta-analysis of diagnostic accuracy of cell-free fetal DNA–based non-invasive prenatal test demonstrated by sensitivity and specificity ratio of common tests ^37^.

Test	Sensitivity	Specificity
Fetal sex	0.989 (95% CI 0.980–0.994)	0.996 (95% CI 0.989–0.998)
Rhesus D	0.993 (95% CI 0.982–0.997)	0.984 (95% CI 0.964–0.993)
Trisomy 21	0.994 (95% CI 0.983–0.998)	0.999 (95% CI 0.999–1.000)
Trisomy 18	0.977 (95% CI 0.952–0.989)	0.999 (95% CI 0.998–1.000)
Trisomy 13	0.906 (95% CI 0.823–0.958)	1.00 (95% CI 0.999–0.100)
Monosomy X	0.929 (95% CI 0.741–0.984)	0.999 (95% CI 0.995–0.999)

CI, confidence interval.

The introduction of NIPT led to a decrease in invasive prenatal diagnostic procedures, but some authors suggest that it also had negative consequences ^41^. Beaudet suggests that it has caused fewer cases of serious deletion syndromes to be detected, resulting in an increased number of births of infants with severe disabilities ^42^. Moreover, a reduction in the number of invasive procedures performed reduces practice opportunities for clinicians, leading to significantly higher miscarriage rates ^43^. Insufficient practice also affects the quality of invasive procedure with more likely side effects, such as infections and fetal loss. In particular, the risks of miscarriage have been estimated to be 0.11% (95% confidence interval [CI] −0.04 to 0.26%) for amniocentesis and 0.22% (95% CI −0.71 to 1.16%) for CVS, according to the systematic review of reported studies in the period of 2000 to 2014 ^44^. Later review of studies in the period of 2000 to 2017 indicated higher risks of miscarriage—0.35% (95% CI 0.07 to 0.63%) and 0.35% (95% CI −0.31 to 1.00%)—for amniocentesis and CVS, respectively ^45^. If this tendency continues, the training and maintaining of skillful clinicians will be a challenge for future prenatal care.

## Fetal cell-based approach

**Figure 2.**
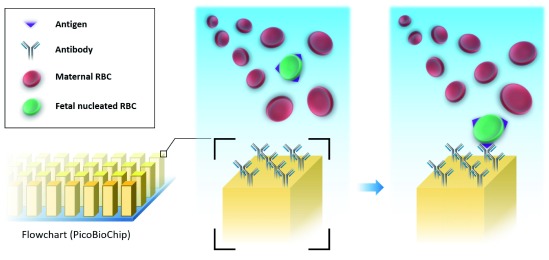
Scheme of silicon-based nanostructured microfluidic platform (Cell Reveal™). The microfluidic device is coated with antibodies which can bind the corresponding antigens on the surface of circulating fetal nucleated red blood cells (RBCs). By this method, fetal cells can be separated from other components of whole maternal blood.

After years of oblivion, several studies recently highlighted the non-invasive analysis of the fetal cells extracted from maternal circulation. It is due to recent advances in single-cell genomics, which opens up opportunities for prenatal screening. Attention in this area has focused on fetal nucleated red blood cells (nRBCs) and trophoblasts. Fetal nRBCs have been the most commonly targeted cell type, however, owing to low-specificity markers and low concentration, trophoblasts have gained more attention ^46^. The value of cell-based non-invasive prenatal diagnosis (cbNIPD) is that limitations of cffDNA-based NIPT can be overcome. In an effort to prevent the problems with fetoplacental mosaicism, Huang *et al*. propose a novel silicon-based nanostructured microfluidic platform (Cell Reveal™) for capturing circulating fetal nRBCs and extravillous cytotrophoblasts for cbNIPD ^47^. This method uses a microfluidic device coated with antibodies which can capture the corresponding antigens on the targeted cells ( Figure 2). The nRBCs isolated through this platform were confirmed to be of fetal, not placental, origin by short tandem repeat analysis, fluorescence *in situ* hybridization, array comparative genomic hybridization, and NGS ^47^. Although the cell-based approach has limitations, it has been shown that individual fetal cells can be isolated from maternal circulation and that their pure fetal DNA can be used for the detection of copy number abnormalities of at least 1 Mb by low-coverage NGS. Analysis of the fetal genome at a higher resolution would allow increased accuracy and improved positive and negative predictive values compared with cfDNA-based NIPT in the detection of microdeletion syndromes ^48^.

## Bioinformatics

Frequent use of NGS led to the production of a large amount of genomic data which need to be processed, stored, analyzed, or transferred (or a combination of these). This maintenance is a challenge for bioinformatics and is the reason why the discipline became an essential part of the modern clinical laboratory. For the acquisition of genetic data from NIPT, two primary massively parallel sequencing approaches are currently available: shotgun sequencing for sequencing of the whole genome and targeted sequencing for sequencing of specific genomic regions of interest ^34^. There are many stages of NGS data analysis where bioinformatics takes place (for example, sequence generation, alignment, and detection of genomic variation) ^49^. Downstream analysis of cfDNA requires bioinformatic algorithms, many of which are based on detection and quantification of imbalances in allelic count, regional genomic representation, or size distribution (or a combination of these) ^50^. Moreover, with the *in silico* bioinformatic approach, it is possible to significantly increase the accuracy and specificity of genetic tests without additional investment for labware ^33, 51^. This highlights the importance and potential of bioinformatics in current NIPT.

Bioinformatic analysis typically consists of two interconnected steps: detecting structural variation and calculating the proportion of fetal fragments in sequenced genomic mixture, called fetal fraction. Traditionally used methods are based on the abundance of sequenced DNA fragments from the affected chromosome. Standard z-score method ^52^ may be refined by fine selection of reference chromosomes ^53^, elimination of long fragments predominantly of maternal origin ^51^, and correction of laboratory-induced bias ^54– 56^ that depart from the observed draw of sequenced reads from ideal uniform distribution ^57^. There is also an emerging field of methods that detect structural aberrations on the basis of diversions of fragment length distributions ^15^. Additionally, count-based and length-based scores may be combined to achieve better separation between euploid and aneuploid samples ^58, 59^ and reduction of false-positive and uninformative predictions ^33, 60^.

Even though count-based methods proved to be highly accurate in routine testing, chromosomal counts alone are not sufficient to determine fetal fraction in cases of pregnancies with female fetuses ^61^ that have the same karyotype as the mother. General methods therefore exploit other characteristics that differ between maternal and fetal DNA fragments, as an uneven distribution of fetal fragments over genome ^62^. Specific deviation has also been observed in regions influenced by packaging of DNA in nucleosomes ^63^. Alternately, fragment length distributions may be used but with lower precision ^15^. Although general methods do not achieve the precision of count-based methods ^64^, their combination along with additional patient attributes such as gestational age and body mass index of the mother ^65^ may further improve their predictions. Single-nucleotide polymorphism (SNP)-based approaches that determine a source of each fragment on the basis of known genotypes of the parents ^66, 67^ would further revolutionize testing in the coming era of genomic biobanking.

## Epigenetics

DNA methylation is a key biological factor that epigenetically regulates the development and function of placenta by gene repression, gene activation, splicing regulation, nucleosome positioning, and the recruitment of transcription factors ^68, 69^. It is known that the placenta plays a crucial role in the normal development of the fetus during pregnancy. Aberrations in placental DNA methylome led to abnormal expression of affected genes and are associated with developmental defects of the fetus ^68^. Thus, current NIPT research is interested in the analysis of placental DNA methylation status ^70^. Whole-genome massively parallel bisulfite sequencing enables clinicians to non-invasively analyze the placental methylome from maternal circulation ^71^. The determination of methylation status is based on treating the DNA with sodium bisulfite, which results in the conversion of unmethylated cytosine into uracil while methylated cytosine remains unchanged ^50^.

The approach of plasma DNA tissue mapping based on the fact that different tissues exhibit different DNA methylation patterns can be used to deduce the origin of cfDNA fragments ^72^. This advantage has great potential to override NIPT limitations caused by maternal malignancy. It means that bisulfite sequencing can be used to differentiate between the origin of fetal-derived cfDNA and tumor-derived cfDNA and thus avoid the false-positive result of NIPT analysis ^73^. However, this advantage also brings the ethical question of how to handle incidental findings (for example, maternal malignancy) ^74^.

To perform whole-genome NGS methylomic analysis, it is necessary to use corresponding bioinformatic software. For example, Methy-Pipe is an integrated bioinformatic pipeline for whole-genome bisulfite sequencing data analysis. This tool allows data pre-processing, sequence alignment, and downstream methylation data analysis, including basic statistics and sequencing quality report, calculation of methylation level, identification of differentially methylated regions for paired samples, annotation and visualization of methylation data for data mining, and easy interpretation ^75^. However, high-resolution whole-genome reconstruction of the placental methylome in a non-invasive manner is still challenging. In an effort to reconstruct the whole fetal/placental methylome, Sun *et al*. propose a novel algorithm called FEMER (fetal methylome reconstructor) ^70^. According to the authors, this approach provides a high‐quality view of the placental methylome from the maternal plasma and might accelerate potential clinical applications ^70^.

An approach that is equivalent to NGS and that could be effective for NIPT of trisomy 21 in pregnant women uses methylated DNA immunoprecipitation combined with quantitative polymerase chain reaction ^76^. This method is based on the selection of fetal–maternal differentially methylated regions, which are used to enrich and assess the fetal DNA ratio ^77^. Statistical evaluation of diagnostic efficiency for trisomy 21 showed 100% specificity and 100% sensitivity of this methodology ^78^. Another validation study showed 99.2% specificity (95% CI 95.62 to 99.98%) and 100% sensitivity (95% CI 92.89 to 100.00%) ^79^. The main advantage of this method is that, in comparison with the NGS, this approach uses equipment that is available in most genetic diagnostic laboratories and is technically easier and less expensive ^77^.

## Conclusions

The current trend in prenatal testing can be characterized by a massive move from invasive sampling to using a non-invasive or more precisely less-invasive source in the form of blood. Despite some limitations of cffDNA analysis of pregnant women, it seems obvious that NIPT will replace other methods of screening for chromosomal aberrations. It is important to understand that NIPT does not entirely replace invasive sampling procedures. Positive NIPT findings must be confirmed by diagnostic tests based on an invasive sample source, mainly amniocentesis. This is different in the case of monogenic disorders, where a haplotyping-based approach allows diagnosis without the need for further confirmation. Continuing research and development efforts are focused on overriding the NIPT limitations, increasing the accuracy, and extending tested defects to reach a diagnostic grade of results and thus to avoid the requirement for confirmation by invasive diagnostic procedures. Invasive testing remains an important part of prenatal care. Recent studies show that procedure-associated risks in the case of amniocentesis are very low when it is performed by experienced clinicians. Unfortunately, widespread adoption of NIPT leads to a drop in performed invasive procedures and experience is decreasing. We believe that novel findings and technological progress will transform NIPT from screening to a final diagnostic test.
